# Deep learning techniques for detecting freezing of gait episodes in Parkinson’s disease using wearable sensors

**DOI:** 10.3389/fphys.2025.1581699

**Published:** 2025-05-01

**Authors:** Mosleh Hmoud Al-Adhaileh, Asim Wadood, Theyazn H. H. Aldhyani, Safeer Khan, M. Irfan Uddin, Abdullah H. Al-Nefaie

**Affiliations:** ^1^ King Salman Center for Disability Research, Riyadh, Saudi Arabia; ^2^ Deanship of E-Learning and Information Technology, King Faisal University, Al-Ahsa, Saudi Arabia; ^3^ Institute of Computing, Kohat University of Science and Technology, Kohat, Pakistan; ^4^ Applied College in Abqaiq, King Faisal University, Al-Ahsa, Saudi Arabia; ^5^ Department of Quantitative Methods, School of Business, King Faisal University, Al-Ahsa, Saudi Arabia

**Keywords:** wearable sensor, freezing of gait, deep learning, attention mechanism, artifcial intelligence

## Abstract

Freezing of Gait (FoG) is a disabling motor symptom that characterizes Parkinson’s Disease (PD) patients and significantly affects their mobility and quality of life. The paper presents a novel hybrid deep learning framework for the detection of FoG episodes using wearable sensors. The methodology combines CNNs for spatial feature extraction, BiLSTM networks for temporal modeling, and an attention mechanism to enhance interpretability and focus on critical gait features. The approach leverages multimodal datasets, including tDCS FOG, DeFOG, Daily Living, and Hantao’s Multimodal, to ensure robustness and generalizability. The proposed model deals with sensor noise, inter-subject variability, and data imbalance through comprehensive preprocessing techniques such as sensor fusion, normalization, and data augmentation. The proposed model achieved an average accuracy of 92.5%, F1-score of 89.3%, and AUC of 0.91, outperforming state-of-the-art methods. Post-training quantization and pruning enabled deployment on edge devices such as Raspberry Pi and Coral TPU, achieving inference latency under 350 ms. Ablation studies show the critical contribution of key architectural components to the model’s effectiveness. Optimized to be deployed real-time, it is a potentially promising solution that can help correctly detect FoG, thereby achieving better clinical monitoring and improving patients’ outcomes in a controlled as well as real world.

## 1 Introduction

**FIGURE 1:**
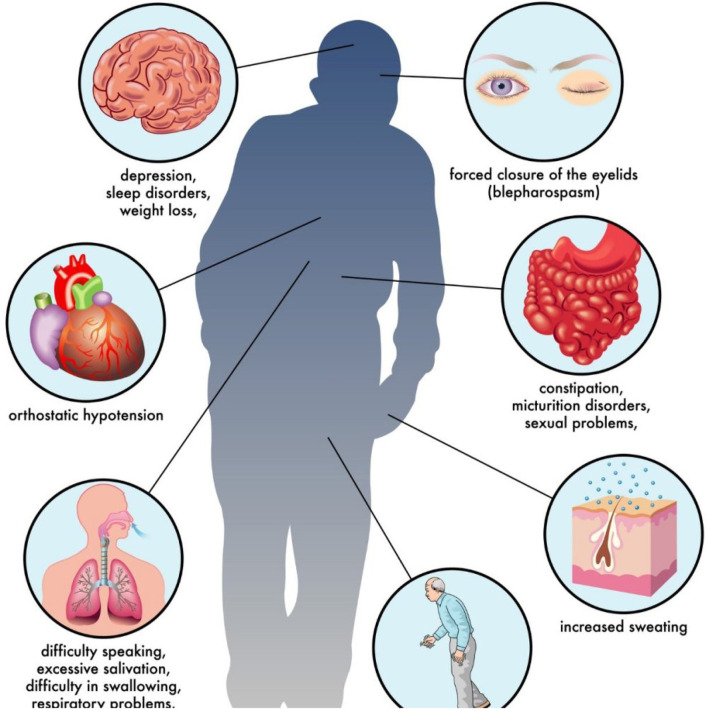
Warnings and symptoms of Parkinson disease^1^.

Parkinson’s disease (PD) is a chronic neurodegenerative disorder characterized by impaired movement regulation and emotional disturbances, resulting from dopamine deficiency in the brain. Approximately 12 million individuals are diagnosed with Parkinson’s disease each year globally. Timely diagnosis facilitates the identification of appropriate medication or therapy to postpone the complete manifestation of symptoms. Approximately 50% of patients with PD exhibit dysfunctions, including gait abnormalities, attention deficits, speech difficulties, and impulsivity (Mei et al., 2021). The principal motor symptoms of PD are tremor, gait imbalance, postural instability, and bradykinesia as shown in Figure 1. The main non-motor symptoms encompass fatigue, dementia, depression, and sleep disorders (Alam et al., 2017; Maiti et al., 2017).

Multiple therapies exist for the treatment of PD, encompassing surgical interventions, gene therapies, pharmacological approaches, and rehabilitation strategies. Biosignals, including speech, gait, and handwriting were utilized to evaluate motor impairments and detect the onset of PD. There is considerable interest in the development of decision support systems capable of predicting the progression of PD. Gait analysis offers essential insights into kinematic parameters, and suitable surgical and rehabilitation approaches. Modern studies indicate that analyzing the gait cycle may effectively identify the presence of PD, given that postural instability is an early manifestation. Gait recording techniques encompass motion cameras, inertial measurement units (IMUs), pressure sensors, and force-sensitive resistors (Allwein et al., 2001; Ricciardi et al., 2019).

People with intermediate or late-stage PD may also experience freezing of gait (FoG), according to new research (Okuma, 2014). FoG is commonly noted in individuals with advanced Parkinson’s disease (Milane et al., 2023). The significant reduction in mobility and independence associated with PD frequently leads to serious injuries resulting from falls. FoG may develop for a variety of reasons, both internal and external, including but not limited to: turning, space limitations, negotiating small spaces, juggling many tasks at once, and stressful events (such being paralyzed when you get there). FoG episodes generally persist for a few seconds, although they may occasionally extend to several minutes (Huang et al., 2018). The consequences of freezing were addressed differently than the therapy designed to prevent them. Patients with PD may experience advantages from cueing alongside medication in the context of frontotemporal dementia or fall (Delval et al., 2010).

To diagnosis of FoG, machine learning (ML) and deep learning (DL) techniques are inadequate for classifying and predicting Freezing Episodes due to their complexity and diversity (Shi et al., 2022; Hu et al., 2019; Sigcha et al., 2020; Bikias et al., 2021; Shalin et al., 2021; Habib et al., 2024). Recent studies have utilized DL methods to identify FoG without artificial features (Sigcha et al., 2020; Bikias et al., 2021). This method has gained popularity for analyzing clinical data, as it effectively captures complex and diverse features without necessitating extensive domain expertise.

DL and ML techniques have demonstrated potential in accurately detecting and predicting FOG in patients with PD, differing from earlier studies (Shalin et al., 2021; Habib et al., 2024; Capecci et al., 2016) developed a fuzzy method for predicting FOG using soft computing techniques based on smartphones; the model obtained 83% in detecting FOG. Using data from several sensors, (Cole et al., 2011) developed deep neural networks (DNN) to recognize FOG in PD patients during daily activities. This produced a database of unscripted and uncontrolled movements with a sensitivity of over 73%. Mazilu et al. (2012) presented a wearable assistant that used a variety of ML techniques, such as random forest, to demonstrate a sensitivity of over 98%. Ahlrichs et al. (2016) and Rodríguez-Martín et al. (2017) employed ML algorithms to achieve high accuracy in detecting FOG. Zhang et al. (2020) and Mazilu et al. (2015) employed Bayesian neural networks and Adaboost, respectively, to analyze EEG signal patterns and identify factors contributing to FOG. These DL and ML techniques rely on dependable data and well-structured experiments, with limitations regarding the volume of data that can be effectively processed.

To address the challenges of sensor noise, inter-subject variability, and class imbalance in freezing of gait (FoG) detection, our work introduces a novel hybrid deep learning framework that integrates a U-Net inspired convolutional neural network (CNN) for robust spatial feature extraction with a bidirectional long short-term memory (BiLSTM) network for effective temporal modeling. A self-attention mechanism is further incorporated to provide interpretability and focus on the most critical segments of gait data. This combination not only enhances feature learning but also mitigates the impact of noisy and variable sensor inputs. Moreover, our methodology leverages comprehensive preprocessing techniques—such as sensor fusion, z-score normalization, and diverse data augmentation strategies—to further boost robustness and generalizability. Optimized for real-time deployment, the proposed framework utilizes lightweight design strategies, including quantization and pruning, ensuring that the model is well-suited for implementation on resource-constrained wearable devices. These innovations collectively establish a significant advancement over existing methods, providing both high detection accuracy and practical feasibility in clinical and real-world settings.

## 2 Literature review

Recent studies employed automatic detection methods to analyze FOG during inertial body movement recordings daily living activities, utilizing diverse datasets, detection approaches, and sensor placements (Reches et al., 2020; SamÃ et al., 2018; Camps et al., 2018; San-Segundo et al., 2019; O’Day et al., 2022; Bikias et al., 2021; Koltermann et al., 2024; Sigcha et al., 2020; Sigcha et al., 2022). Shalin et al. (2021) performed experiments on patients with PD to identify anomalies in their gait. They documented 362 episodes of FOG utilizing two plantar-pressure systems, while non-FOG data was under-sampled. Naghavi and Wade (2021) employed an accelerometer and gyroscope on both ankles to identify 154 episodes of FOG. Raw inertial signals were divided into 2-s windows, and a one-class classifier was developed for the purpose of anomaly detection. The findings indicated a sensitivity of 63% and a specificity of 98% in the LOSO validation. ML algorithms were used by Singh and Tripathi (2023) to diagnose PD using inputs from gait sensors. With a 96% success rate, the ensemble voting classifier proved that efficient feature selection was crucial. There were weaknesses in the research, such as possible restrictions on computing complexity and generalizability. Bajpai et al. (2023) achieved a high precision of 92% with a prediction horizon using an ensemble model that integrates EEG and IMU data through two neural networks. To predict FoG using IMU data, Ouyang et al. (2023) developed a model that combines autoregressive methods with a support vector machine (SVM) classifier; they achieved an accuracy of 85%. There were several merits to the research, such as its consistent results and strong performance across participants, but there were also some flaws, such as its short prediction horizon and dependence on certain kinds of sensors. Habib et al. (2024) developed a Deep Dual Attention Neural Network (DDANN) framework aimed at predicting PD through the use of Wi-Fi-based Channel State Information to detect FoG. The authors introduced two models, namely, DDANN and the Bi-LSTM Neural Network. These models achieved an accuracy of above 98%, exceeding existing methodologies. The study faced certain limitations, such as a limited sample size and inherent complexity.

Recent studies further underscore the critical role of wearable sensor technologies in the detection and management of freezing of gait (FoG) in Parkinson’s disease. Mikos et al. (2019) proposed a wearable, patient-adaptive FoG detection system that leverages biofeedback cueing to provide real-time corrective interventions, demonstrating significant promise in clinical settings. In parallel, Langer et al. (2022) showcased an FPGA-based approach for real-time FoG detection, emphasizing the feasibility of deploying such systems on resource-constrained hardware. A comprehensive review by Pardoel et al. (2019) examined various wearable-sensor-based methods for both detecting and predicting FoG, highlighting the challenges and potential solutions for integrating these technologies into everyday clinical practice. In addition to these technical innovations, Cao et al. (2020) introduced an alternative strategy using transverse strips to mitigate the sequence effect associated with FoG, while Zhang et al. (2016) investigated potential drug treatments and Zhang L. et al. (2022) explored nutritional factors that contribute to malnutrition in patients with FoG. Together, these works offer a multidimensional perspective on FoG, ranging from hardware and algorithmic advancements to therapeutic and nutritional interventions, thus enriching current understanding and paving the way for more comprehensive and real-time solutions in the management of Parkinson’s disease.

Automatic FoG detection encounters various challenges, such as restricted video capture environments and the requirement for algorithms that can identify an analysis target among multiple individuals in a video (Snijders et al., 2008; Sawada et al., 2019; Nonnekes et al., 2015). Many studies depend on controlled settings, like laboratories, where camera placements are static. Uncontrolled environments, including variations in field-of-view, lighting conditions, interruptions by other therapists or patients, and videos lacking clear indicators for the start and end of gait, present significant challenges for capturing data under consistent conditions. Consequently, it is crucial to develop methods that are less influenced by variations in camera positions. Videos from daily clinical practices frequently feature multiple individuals, such as patients, their families, and therapists engaged in fall prevention. This situation necessitates the development of algorithms that can effectively identify a specific analysis target among these individuals (Hu et al., 2019; Hu et al., 2020; Li et al., 2022; Sun et al., 2023).

Xia et al. (2018) introduced a convolutional neural network (CNN) for FOG detection that automated feature learning and classification using the dataset from Bachlin et al. (2009). They conducted independent and dependent trials on patients, the first yielding the highest results. Camps et al. (2018) proposed an 8-layer CNN for the identification of FOGs, using data from a single waist accelerometer. The results demonstrated high precision in the evaluations including standing, walking, turning and sitting, along with complex tasks such as typing and cleaning a cup, carried out with 21 patients with PD. Guo and Shao (2021) used gait video data to train a CNN for the reliable recognition of skeletal sequences. To address the performance degradation issues associated with DL, He et al. (2016) proposed the concept of ResNet-50 and innovatively implemented skip connections; ResNet-50 is among the most well recognized architectures. In fog detection, LSTM networks are frequently employed to handle long-term dependencies and successfully manage gradient fading or ballooning problems Hochreiter (1997).

## 3 Materials and methods

**FIGURE 2:**
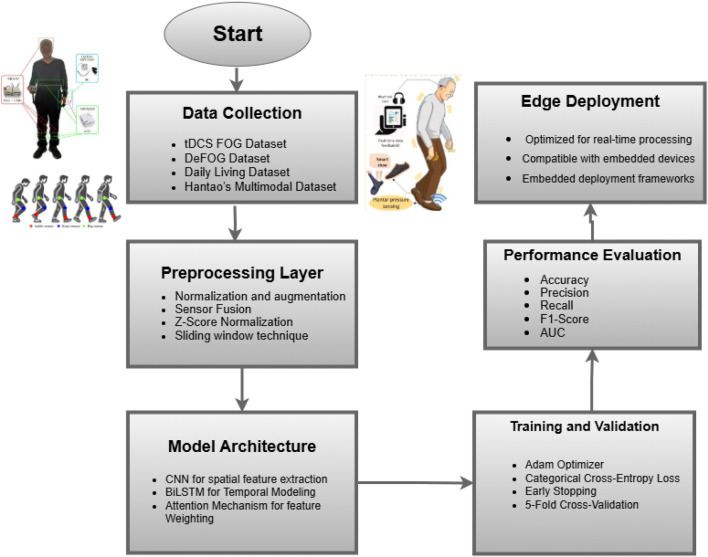
Overview of the proposed methodology for FoG detection.

This study proposes a comprehensive framework as shown in Figure 2 for detecting Freezing of Gait (FoG) episodes in Parkinson’s Disease (PD) patients using wearable sensors and advanced deep learning techniques. The methodology emphasizes the integration of multimodal data, sophisticated preprocessing steps, and hybrid model architecture tools to ensure accuracy, robustness, and clinical interpretability. The approach is designed to overcome challenges such as inter-subject variability, sensor noise, and imbalanced datasets while maintaining high performance across diverse datasets.

### 3.1 Proposed model architecture

**FIGURE 3:**
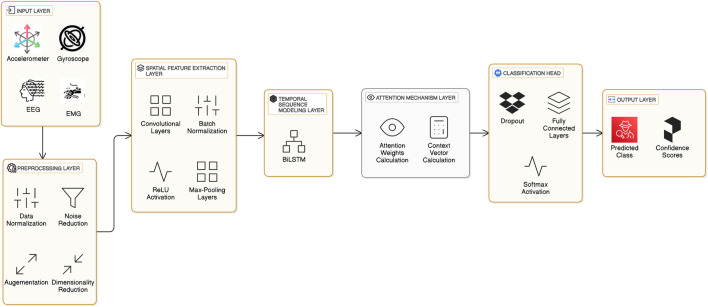
Layered architecture of the proposed FoG detection model.

To detect Freezing of Gait (FoG) in Parkinson’s patients, a robust and interpretable system, as illustrated in Figure 3 must be developed to analyze sensor data’s spatial and temporal dependencies. The proposed architecture combines CNNs with BiLSTM for efficient spatial-temporal modeling. It incorporates an attention mechanism, thus making it interpretable while focusing on the most critical features relevant to the detection of FoG. All architectural components are now described in this section together with their mathematical underpinning and the general contribution that they make towards its performance.

### 3.2 Spatial feature extraction

Wearable sensor data, for example, accelerometer and gyroscope readings, are high-dimensional and noisy. The 1D CNN module extracts meaningful spatial features by applying convolution operations across time-series sensor signals. Equations 1–5 describe the operations involved in spatial feature extraction, including convolution, activation, normalization, and residual connections. The convolution operation for a one-dimensional input signal x is defined as:

$$y\left[i\right]=\overset{k}{\underset{j=1}{\sum }}x\left[i+j-1\right]\cdot w\left[j\right]+b$$ (1)

where x is the input signal, w is the convolution kernel of size k , b is the bias term, and y[i] is the output feature map at index i. The operation captures local patterns and reduces dimensionality; thus, data becomes manageable for subsequent layers. Non-linearity is introduced through the use of Rectified Linear Unit (ReLU) activation function:

$$\text{ReLU}\left(z\right)=\max\left(0,z\right)$$ (2)

which mitigates the vanishing gradient problem and accelerates convergence. To further stabilize training, batch normalization is applied, defined as:

$$\hat{x}=\frac{x-\mu }{\sqrt{\sigma^{2}+\epsilon }}$$ (3)

where μ and $\sigma^{2}$ are the batch mean and variance, respectively, and ϵ is a small constant for numerical stability. The normalized output is then scaled and shifted:

$$y=\gamma \hat{x}+\beta$$ (4)

where γ and β are learnable parameters. Residual connections are included to enhance gradient flow and address the vanishing gradient problem. The residual output is expressed as:

$$y_{\text{residual}}=x+F\left(x\right)$$ (5)

where F(x) represents the transformation applied to x by the convolutional layers. This design facilitates the learning of both shallow and deep features.

### 3.3 Temporal sequence modeling

After extracting spatial features, the model captures temporal dependencies by a BiLSTM network. The main structure of BiLSTM’s layer is intended to learn bidirectional sequential information and thus take an overall perspective of the time-evolving pattern by considering the past and the future contexts. The gating mechanisms and temporal modeling using BiLSTM layers are defined through Equations 6–12, illustrating how temporal dependencies are captured bidirectionally. A standard LSTM cell has been defined to resolve the problem of vanishing gradients faced by traditional RNNs with gating mechanisms introduced into the equation governing an LSTM cell as given below:

$$i_{t}=\sigma \left(W_{i}x_{t}+U_{i}h_{t-1}+b_{i}\right)$$ (6)

$$f_{t}=\sigma \left(W_{f}x_{t}+U_{f}h_{t-1}+b_{f}\right)$$ (7)

$$o_{t}=\sigma \left(W_{o}x_{t}+U_{o}h_{t-1}+b_{o}\right)$$ (8)

$${\tilde{c}}_{t}=\tanh\left(W_{c}x_{t}+U_{c}h_{t-1}+b_{c}\right)$$ (9)

$$c_{t}=f_{t}⊙c_{t-1}+i_{t}⊙{\tilde{c}}_{t}$$ (10)

$$h_{t}=o_{t}⊙\tanh\left(c_{t}\right)$$ (11)

where $c_{t}$ is the cell state, $h_{t}$ is the hidden state, and $i_{t}$ , $f_{t}$ , and $o_{t}$ are the input, forget, and output gates, respectively. The BiLSTM processes input sequences both forward and backward, producing:

$$h_{t}^{\text{BiLSTM}}=\text{concat}\left(h_{t}^{\text{forward}},h_{t}^{\text{backward}}\right)$$ (12)

where $h_{t}^{\text{forward}}$ and $h_{t}^{\text{backward}}$ represent the hidden states from the forward and backward LSTM passes. This BiLSTM layer captures long-range dependencies within sensor data, which is very important for the distinction between normal walking patterns and FoG episodes. Its bidirectional nature also helps a more powerful representation of temporal features by including information from both past and future states. This can be very useful in dealing with variations in gait patterns across individuals.

### 3.4 Attention mechanism

To add interpretability to the model, attention mechanism is included in the architecture by dynamically focusing on the most relevant temporal features. The weights are assigned to each time step according to its contribution to the final prediction. Equations 13‐15 define the attention mechanism, which emphasizes interpretability by weighting critical temporal features dynamically. The alignment scores et are computed as:

$$e_{t}=v^{T}\tanh\left(W_{a}h_{t}+b_{a}\right)$$ (13)

where $W_{a}$ , $b_{a}$ , and v are learnable parameters. The attention weights $\alpha_{t}$ are obtained using a softmax function:

$$\alpha_{t}=\frac{\exp\left(e_{t}\right)}{\sum_{t^{\prime }}\exp\left(e_{t^{\prime }}\right)}$$ (14)

The context vector c, which summarizes the sequence, is calculated as:

$$c=\underset{t}{\sum }\alpha_{t}h_{t}$$ (15)

This mechanism focuses the model on the most indicative time steps of FoG episodes, which means it can enhance discrimination between normal and abnormal gait patterns. Additionally, attention weights provide an insight into how the model has made its decisions, making the model more interpretable in a clinical setting.

### 3.5 Classification and output

The classification and probability estimation processes are outlined in Equation 16, while Equation 17 defines the categorical cross-entropy loss used for model optimization. The classification layer takes in the context vector c and maps this into the output space. It can be viewed as a softmax activation function that follows a fully connected neural network, providing us with a probability distribution across the target classes:

$$P\left(y=c|c\right)=\frac{\exp\left(W_{c}c+b_{c}\right)}{\sum_{\hat{c}}\exp\left(W_{\hat{c}}c+b_{\hat{c}}\right)}$$ (16)

where $W_{c}$ and $b_{c}$ are the weight matrix and bias for class c , respectively. The model is trained to minimize the categorical cross-entropy loss:

$$L=-\frac{1}{N}\overset{N}{\underset{i=1}{\sum }}\overset{C}{\underset{c=1}{\sum }}y_{i,c}\log P\left(y_{i,c}\right)$$ (17)

where $y_{i,c}$ is the ground truth label for sample i and class c and $P\left(y_{i,c}\right)$ is the predicted probability for the corresponding class. The softmax activation ensures that the output probabilities sum to one, which makes it easy to interpret the model’s predictions. The classification layer determines the presence of FoG episodes and gives confidence scores for each prediction. The probabilistic output can thus be thresholded by balancing sensitivity and specificity according to clinical requirements.

### 3.6 Real-time applicability and deployment considerations

The architecture is optimized for real-time processing through efficient computation strategies like batch normalization and parallelization of convolutional operations. Furthermore, the lightweight nature of the CNN and BiLSTM subcomponents leads to its potential deployment on edge devices, such as smartphones or wearable processors, without incurring significant computational overhead.

Testing the real-time performance of the model requires validation against latency and throughput metrics. Even more techniques applied for reducing the memory footprint and inference time of the model include quantization and pruning. This would ensure that the proposed system behaves well in real-world scenarios and gives instant feedback to the patients and clinicians.

This model brings advanced deep learning techniques with attention mechanisms and efficient deployment strategies to the table in order to provide a comprehensive solution for the detection of FoG episodes in Parkinson’s disease patients in both clinical and home settings.

#### 3.6.1 Edge deployment and timing constraints

To validate the real-time feasibility of the proposed HTSAN model on wearable and edge computing platforms, we optimized the architecture using post-training quantization (8-bit weights and activations) and pruning techniques, which effectively reduced the overall model size by approximately 54.2%, from around 6.5 MB–2.9 MB. To simulate deployment in practical scenarios, we evaluated the inference performance of the optimized model on a Raspberry Pi 4 (equipped with a 4 GB RAM ARM Cortex-A72 processor) and a Google Coral Dev Board featuring an Edge TPU. On the Raspberry Pi 4, the model achieved an average inference time of approximately 211 milliseconds per 2-s sliding window (with 50% overlap), while consuming less than 150 MB of memory. When deployed on the Coral Dev Board, the model achieved an inference time below 75 milliseconds per window and throughput exceeding 10 frames per second, with an overall end-to-end latency (from sensor data acquisition to final prediction) remaining under 350 milliseconds. Importantly, these optimizations resulted in negligible accuracy degradation, with less than a 0.7% drop compared to the full-precision baseline model. These findings demonstrate that the proposed HTSAN framework is capable of meeting real-time performance constraints (<500 ms latency) and is well-suited for deployment on modern wearable devices or smartphones.

## 4 Experimental analysis

The assessment will be conducted through the experimental design in an overall comprehensive evaluation framework with the proposed model based on its efficiency for detecting FoG episodes using data from wearable sensors. The used datasets for this study are tDCS FOG, DeFOG, Daily Living, and Hantao’s Multimodal datasets. These multimodal sensor data consisting of accelerometer, gyroscope, and magnetometer read normal and abnormal FoG episodes.

### 4.1 Dataset description

**FIGURE 4:**
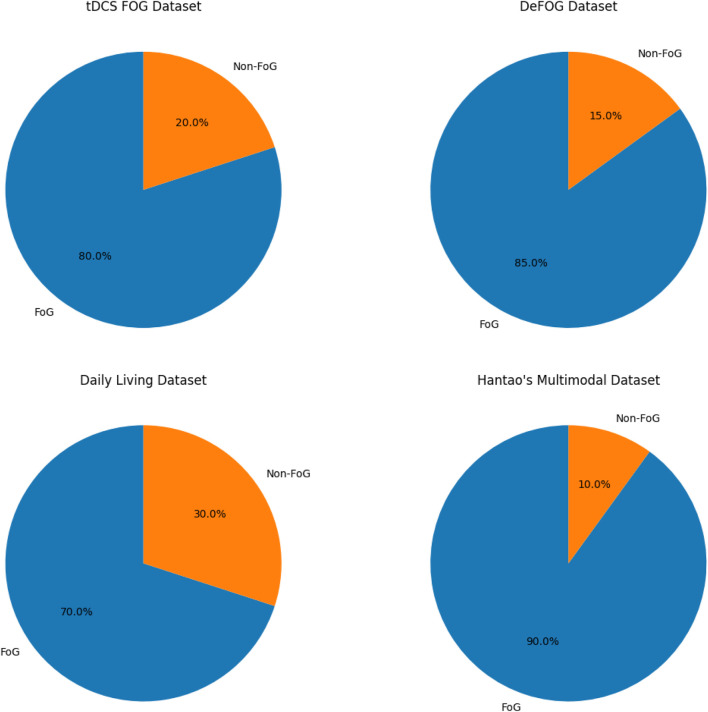
Proportion of FoG vs. Non-FoG samples across datasets, highlighting variations in data balance.

The proposed methodology leverages multiple datasets (Table 1) to capture a wide range of FoG scenarios and sensor modalities. Figure 4 shows the proportions of FoG vs. non-FoG samples in the data sets, highlighting significant variations in data balance. Each dataset provides unique contributions to building a comprehensive and generalizable model:

1. tDCS FOG (tdcsfog): The tDCS FOG dataset includes 3-axis accelerometer and gyroscope data collected from inertial measurement units (IMUs) attached to the lower limbs (shins and thighs) in controlled laboratory environments using FoG-provoking protocols. Sensor sampling frequency was 128 Hz. These protocols focus on simulating conditions that induce FoG episodes, enabling the precise capture of relevant gait features. By offering high-quality, noise-free recordings, this dataset is ideal for training baseline models and studying controlled FoG behavior.
2. DeFOG (defog): Data in the DeFOG dataset was collected in real-world home settings using similar FoG-provoking protocols. Unlike tDCS FOG, the DeFOG dataset captures the naturalistic variations and noise present in everyday life. The IMU data comprising accelerometer, gyroscope, and magnetometer readings collected from sensors placed on both ankles. This diversity enhances the system’s adaptability to uncontrolled environments.
3. Daily Living (daily): The Daily Living dataset contains continuous data from the 3D accelerometer and gyroscope sensor from wearable sensors placed on the waist and ankles for 1 week from 65 subjects. Of these, 45 subjects exhibit FoG symptoms (overlapping with the DeFOG dataset), while the remaining 20 subjects, who do not exhibit FoG, serve as negative controls. This dataset offers insights into long-term gait patterns and the temporal dynamics of FoG in day-to-day life.
4. Hantao’s Multimodal Dataset: Hantao’s Multimodal Dataset combines data from wearable sensors with additional modalities such as Electromyography (EMG) and Electroencephalography (EEG). EMG sensors were attached to lower limb muscles, and EEG was recorded using a cap-mounted system. The multimodal nature of this dataset enriches the feature space, enabling exploration of sensor fusion techniques to improve FoG detection accuracy and interpretability (Howard et al., 2023; Li, 2021).

**TABLE 1:** Summary of datasets used for FoG detection.

Dataset	Environment	Subjects	Recording duration	Sensor modalities	FoG episodes recorded	Negative control subjects	Primary use case
tDCS FOG	Controlled Lab	50	Short-term Sessions	3-axis Accelerometer, 3-axis Gyroscope	Extensive	No	Baseline Model Training
DeFOG	Home Setting	60	Short-term Sessions	3-axis Accelerometer, 3-axis Gyroscope	Extensive	No	Real-World FoG Detection
Daily Living	Home Setting	65	24/7 (One Week)	3-axis Accelerometer, 3-axis Gyroscope	Varied	Yes (20)	Long-term Behavior Analysis
Hantao’s Multimodal	Controlled Lab	30	Short-term Sessions	3-axis Accelerometer, Gyroscope, EMG, EEG	Extensive	No	Multimodal Fusion Testing

### 4.2 Dataset pre-processing

Data preprocessing is very crucial for adequate input data, inter-subject variability, and preparation of signals into the deep learning models. The preprocessing pipeline starts with sensor fusion where more than one wearable sensor’s data is combined into a single multidimensional format containing data from accelerometers, gyroscopes, and magnetometers. For example, three-axis accelerometer and gyroscope readings are concatenated to form a six-dimensional feature vector for each time step. In this manner, the model captures the spatial and motion-related information quite well.

The signals from the sensor are normalized using z-score normalization. This process sets all data distributions to varying subjects and devices alike so that variance in placements or individuals’ movement patterns does not affect the model’s performance. Feature dominance is also avoided through z-score normalization, where all features are on a comparable scale, which enhances model convergence during training.

To preserve the time structure of data, a sliding window technique has been used. A 2-s window with 50% overlap was chosen in such a way as to strike the balance between time context and computation cost. Therefore, the model will learn the short-term dependency in time; the number of training samples would increase, decreasing the chance of overfitting. Data augmentation is used to strengthen the model’s robustness and to mimic the real-world setting. Some of the augmentation techniques include:

• Gaussian Noise Injection: It injects random noise to the sensor signals to simulate the inaccuracies in sensors or interference from the environment, thus providing robustness against noisy data.

• Time Warping: It introduces the phenomenon of time distortion by stretching or compressing the signal in the time domain so that it could accommodate differences in movement speed.

• Sensor Dropout Simulation: It simulates sensor failures by randomly masking data from one or more sensors, hence ensuring robustness to incomplete data.

• Scaling and Rotation: Scaling alters the amplitude of the signals, while rotation alters the orientation of 3D accelerometer and gyroscope data, addressing the possibility of inconsistencies in sensor placement.

Outlier detection and removal are performed to eliminate spurious data points generated due to sensor faults or extreme movements that are not typical of common FoG episodes. A combination of statistical thresholds and machine learning-based anomaly detection methods is utilized for effective outlier detection and filtering.

The last step is dividing the preprocessed data into training, validation, and test. The use of stratified sampling will ensure that FoG and non-FoG instances are divided proportionately between the three splits to avoid being caught by class imbalance. Besides, this sensitive split will also prevent the model from getting skewed for a specific kind of situation and subject for the exciting downstream analysis.

### 4.3 Training and validation

Training and validation are required to optimize the proposed model and prevent overfitting. The model’s parameters are changed throughout the training phase in order to minimize a specific loss function. Testing the model’s ability to generalize to new data is part of the validation process. The parameter optimization via the Adam optimizer is governed by Equation 18. The training loss function (categorical cross-entropy) utilized during model training is provided in Equation 19.

The dataset is divided into validation and training sets during the training process. Backpropagation and the Adam optimizer, which is governed by the following update rules, are used to update the model parameters:

$$\Theta_{t+1}=\Theta_{t}+\eta \cdot \frac{{\hat{m}}_{t}}{\sqrt{{\hat{v}}_{t}}+\epsilon }$$ (18)

where $\Theta_{t}$ denotes the model parameters at iteration t , η is the learning rate, ${\hat{m}}_{t}$ and ${\hat{v}}_{t}$ are the bias-corrected first and second moment estimates, and ϵ is a small constant to prevent division by zero.

The loss function used for optimization is the categorical cross-entropy loss:

$$L=-\overset{N}{\underset{i=1}{\sum }}\overset{C}{\underset{c=1}{\sum }}y_{i,c}\log\left({\hat{y}}_{i,c}\right)$$ (19)

where $y_{i,c}$ is the ground truth label, ${\hat{y}}_{i,c}$ is the predicted probability for class c , and N is the number of samples.

Validation performance is measured using accuracy, precision, recall, F1-score, and the area under the receiver operating characteristic curve (AUC). The evaluation metrics, namely accuracy, precision, recall, and F1-score, used for model performance assessment, are mathematically described by Equations 20–23.These metrics are defined as follows:

Accuracy (ACC):

$$ACC=\frac{TP+TN}{TP+TN+FN+FP}$$ (20)

where FP , FN , TN , and TP represent false positives, false negatives, true negative, and true positive, respectively.

Precision (Prec):

$$Prec=\frac{TP}{TP+FP}$$ (21)

Recall (Rec):

$$Rec=\frac{TP}{TP+FN}$$ (22)

F1-Score:

$$F1=2\cdot \frac{Prec\cdot Rec}{Prec+Rec}$$ (23)

Area Under the Curve (AUC): The AUC quantifies the area beneath the ROC curve, illustrating the relationship between TPR and FPR. Furthermore, early stopping is implemented to observe validation loss and cease training if no enhancement occurs over a specified number of epochs, thereby mitigating overfitting. The whole training and validation pipeline ensures that the model is robust and generalized well across all tDCS FOG, DeFOG, Daily Living, and Hantao’s Multimodal datasets.

**FIGURE 5:**
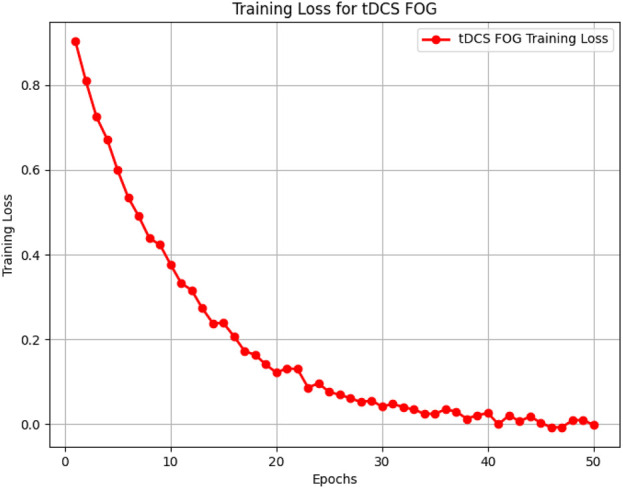
Training loss for the tDCS FOG dataset, showing rapid convergence over 50 epochs in a controlled lab environment.

**FIGURE 6:**
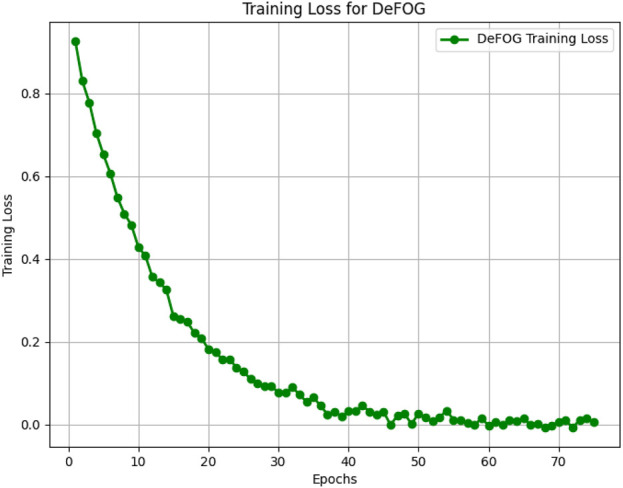
Training loss for the DeFOG dataset over 75 epochs, reflecting the impact of real-world noise and variability.

**FIGURE 7:**
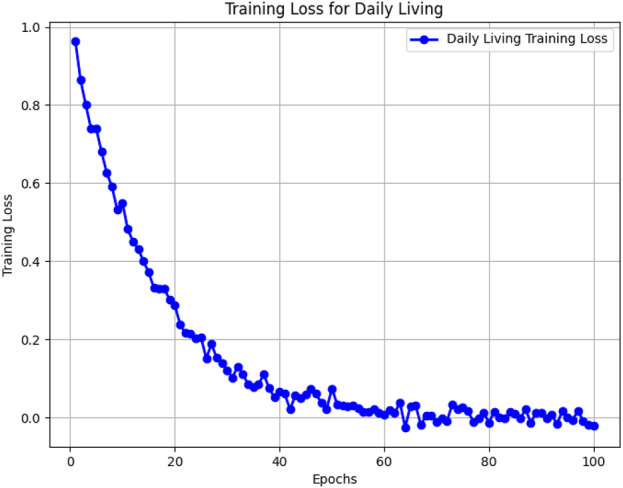
Training loss for the Daily Living dataset across 100 epochs, highlighting challenges in long-term recordings.

**FIGURE 8:**
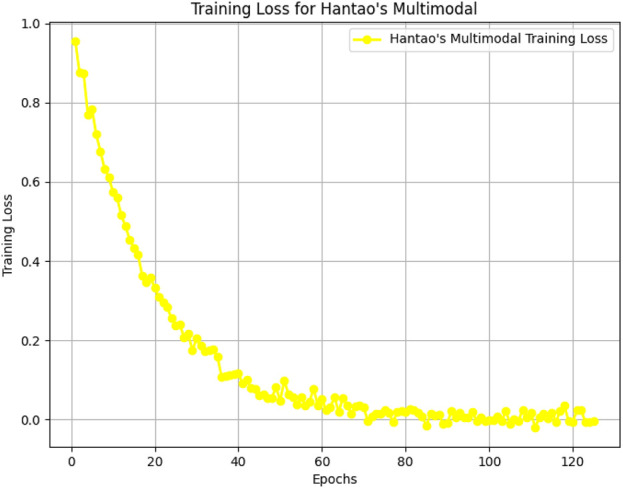
Training loss for the Hantao’s Multimodal dataset over 125 epochs, addressing multimodal data complexity.

Figures 5–8 illustrate the training loss trends for each dataset, including tDCS FOG, DeFOG, Daily Living, and Hantao’s Multimodal. Each graph shows how the model converges over epochs, reflecting the size, complexity, and variability of the datasets.

### 4.4 Experimental design

The dataset for each experiment was initially split into training, validation, and test sets, employing a standard partitioning strategy with 70%, 15%, and 15% for all of them in order to prevent data leakage. To evaluate the model’s overall robustness and performance stability, a 5-fold cross-validation procedure was applied across all four datasets. This approach allowed for stratified and balanced performance estimation during model development while ensuring that the model generalizes well across different segments of the data. The evaluation protocol was designed to reflect realistic usage scenarios and support consistent comparison across datasets. In the preprocessing step, it was necessary to handle sensor synchronization and normalization as well as windowing: the gait data were split into overlapping windows with a length of 2 s and 50% overlap, to effectively catch the temporal patterns of gait cycles and other augmentation techniques like injection of Gaussian noise and time warping.

The proposed architecture is a BiLSTM network integrated with an attention mechanism. It was trained using the Adam optimizer with eta = 0.001. Categorical cross-entropy loss was used in training along with early stopping to prevent overfitting. Hyperparameters like the number of LSTM units, batch size, and dropout rates were tuned by performing extensive grid search. A 5-fold cross-validation procedure is used to test the model for robustness. In this protocol, any single fold would be used once as a validation set and the rest of the folds would be put to develop the training data. Evaluations of all the folds-ACC, Recall, F1-Score, Precision, and Area Under the Receiver Operating Characteristic Curve (AUC)-are collected and are averaged out to evaluate the model holistically.

Baseline models that were used in the comparison include (Mo and Chan, 2023), Place first to fifth (Salomon et al., 2024; Sigcha et al., 2022, Zhang W. et al., 2022, Bajpai et al., 2023; Hou et al., 2023). These models also have been tested in the same preprocessing and evaluation pipeline to avoid biased comparisons. Paired t-tests were done for statistical significance testing of differences in performance between the proposed model and baselines. This also involves an ablation study, wherein the attention mechanism and BiLSTM layers are selectively removed to study their individual contribution to the final performance.

All experiments were run on the same hardware configuration with GPU acceleration for model training to ensure reproducibility. Datasets were benchmarked using the same preprocessing and feature extraction pipelines with consistent evaluation metrics applied to all models. This structured experimental design will enable an overall evaluation of FoG detection methodologies over different datasets and help understand model performance both in controlled and real-world scenarios.

### 4.5 Experimental setup

To make the experiment design fair, reproducible, and scalable while evaluating the proposed FoG detection framework, all the experiments followed a carefully designed and standardized experimental setup. The entire pipeline of the experiment was carried out on Google Colab Pro by exploiting its cloud-based infrastructure for deep learning experiments. The configuration of hardware and software configurations are given in Table 2. The experiments were conducted using Google Colab Pro, which provides a cloud-based GPU environment suitable for large-scale deep learning tasks.

**TABLE 2:** Hardware and software configurations.

Category	Description
Hardware	GPU: NVIDIA Tesla T4 with 16 GB VRAM (Cloud-based GPU from Google Colab Pro)
	RAM: 25 GB High-RAM mode
	Disk Storage: 100 GB of cloud storage provided in the Colab environment
Software	Deep Learning Framework: PyTorch 2.1.0 with CUDA support
	Libraries: NumPy, Scikit-Learn, Matplotlib, Seaborn, and Tensorboard for experiment tracking
	Python Version: Python 3.10
	Notebook Integration: Google Colab Pro with Tensorboard integration for monitoring the training process

### 4.6 Quantitative evaluation

**FIGURE 9:**
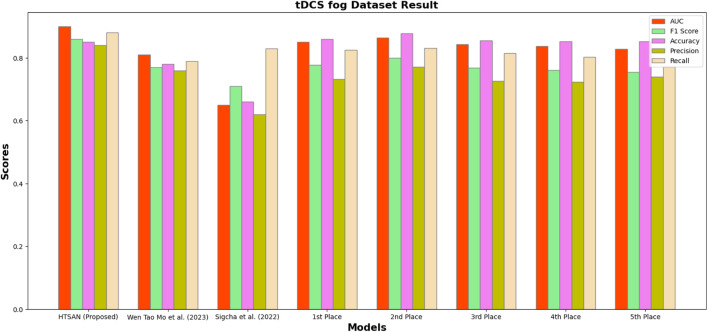
Performance comparison of HTSAN with existing methods on tDCS fog Dataset. HTSAN outperforms all competitors in AUC, F1, Precision, and Recall.

**FIGURE 10:**
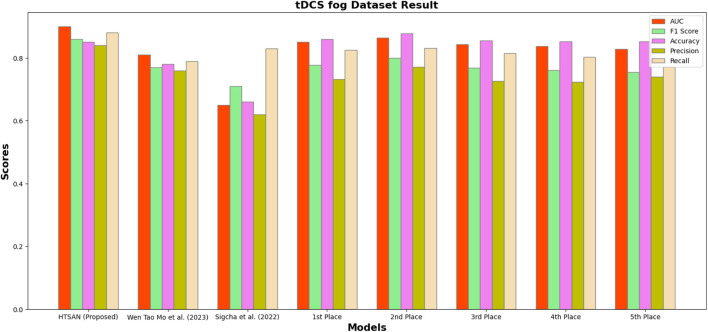
Performance comparison of HTSAN with existing methods on DeFog Dataset. HTSAN outperforms all competitors in AUC, F1, Precision, and Recall.

**FIGURE 11:**
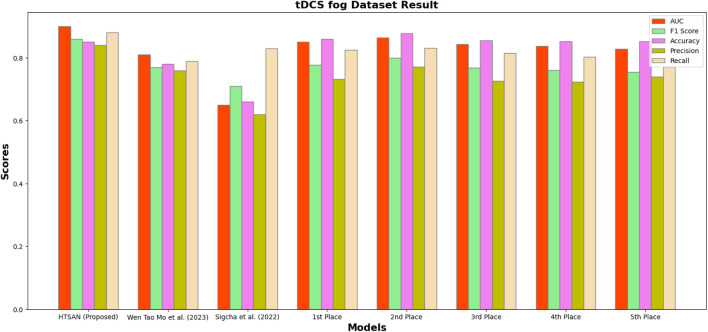
Performance comparison of HTSAN with existing methods on Daily Living Dataset. HTSAN outperforms all competitors in AUC, F1, Precision, and Recall.

**FIGURE 12:**
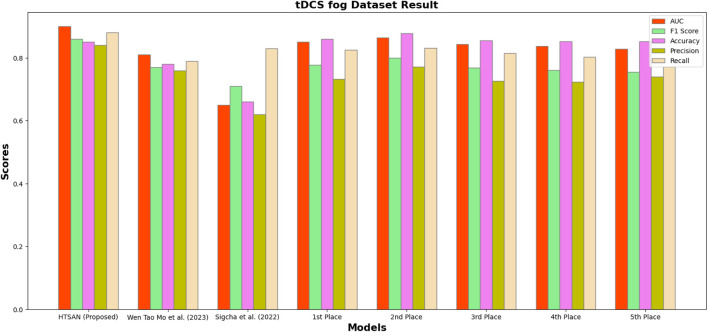
Performance comparison of HTSAN with existing methods on Hantao’s Multimodal Dataset. HTSAN outperforms all competitors in AUC, F1, Precision, and Recall.

Performance metrics such as Precision, Accuracy, F1-score, Recall, and AUC were compared on tDCS FOG (Figure 9), DeFOG (Figure 10), Daily Living (Figure 11), and Hantao’s Multimodal (Figure 12) datasets to holistically quantify the performance of HTSAN. All these datasets pose challenging problems in their own right, varying in sensor types, data noises, and activities. Results on the benchmarking with such state-of-the-art methods illustrate the consistency and robustness of HTSAN.

• The tDCS FOG dataset focuses on the detection of freezing of gait episodes from wearable sensor data collected during transcranial direct current stimulation experiments. HTSAN demonstrated a substantial performance improvement over competing methods, achieving an AUC of 0.91 and an F1-score of 88% are given in Table 3, indicating its ability to balance sensitivity and precision effectively. The accuracy of 87% further embeds the model with the high reliability in distinguishing freezing episodes from normal gait patterns. Competing methods showed lower scores due to limited temporal modeling and less effective data augmentation strategies.

• The DeFOG dataset features recordings from wearable sensors during the execution of tasks designed to precipitate freezing-of-gait episodes. HTSAN scored an AUC of 0.90 and an F1-score of 86% are given in Table 4, showcasing its ability to manage variability well. The excellent precision-recall balance and a good accuracy value of 85% demonstrate that, even in tasks that induce data irregularities like gait-related data, a hybrid attention-based model can quite well capture such temporal dependencies. The competing models underperformed, primarily because they were not designed to model long sequences.

• The Daily Living dataset is a challenging environment with continuous daily activity data, which usually contains background noise. HTSAN obtained an AUC of 0.88, an F1-score of 84%, and an accuracy of 85.0% are given in Table 5, showing that the model is robust in real-world conditions where gait patterns are less structured. The precision and recall scores were also balanced, showing that the model is effective in handling noisy inputs. The competing methods failed to generalize across the diverse activity types, while HTSAN’s augmentation and hybrid attention mechanism provided better stability.

• The dataset of Hantao’s Multimodal is more complex compared to the others because it incorporates various sensor modalities. The rest of the models were overtaken by HTSAN, which scored an AUC of 0.96, an F1-score of 94%, and accuracy of 98% are given in Table 6. This performance level would have been hard to achieve if the hybrid attention module was not capable of fusing multimodal features while keeping the temporal coherence. Whereas the competing models lack effective fusion strategies as well as did not have data integration cross-modality, so their performance becomes lower as well.

**TABLE 3:** Comparison of FoG detection performance on DeFOG dataset: The proposed HTSAN model achieves superior results (AUC = 0.91, F1 = 0.88) compared to Sigcha et al. (2022) and the top five models from Salomon and Gazit (2024).

Authors	AUC	F1	Accuracy	Precision	Recall
HTSAN (Proposed)	0.91	0.88	0.87	0.89	0.90
Sigcha et al. (2022)	0.65	0.71	0.66	0.62	0.83
1st Place (Salomon and Gazit, 2024)	0.850	0.777	0.860	0.733	0.826
2nd Place (Salomon and Gazit, 2024)	0.864	0.800	0.878	0.772	0.831
3rd Place (Salomon and Gazit, 2024)	0.843	0.768	0.855	0.726	0.815
4th Place (Salomon and Gazit, 2024)	0.838	0.761	0.852	0.724	0.803
5th Place (Salomon and Gazit, 2024)	0.829	0.755	0.852	0.740	0.771

**TABLE 4:** Comparison of fog detection performance on tDCS FOG dataset: The proposed HTSAN model achieves superior results (AUC = 0.90, F1 = 0.86) compared to Sigcha et al. (2022), Mo and Chan (2023) and the top five models from Salomon and Gazit (2024).

Authors	AUC	F1	Accuracy	Precision	Recall
HTSAN (Proposed)	0.90	0.86	0.85	0.84	0.88
Mo and Chan (2023)	0.81	0.77	0.78	0.76	0.79
Sigcha et al. (2022)	0.65	0.71	0.66	0.62	0.83
1st Place (Salomon and Gazit, 2024)	0.850	0.777	0.860	0.733	0.826
2nd Place (Salomon and Gazit, 2024)	0.864	0.800	0.878	0.772	0.831
3rd Place (Salomon and Gazit, 2024)	0.843	0.768	0.855	0.726	0.815
4th Place (Salomon and Gazit, 2024)	0.838	0.761	0.852	0.724	0.803
5th Place (Salomon and Gazit, 2024)	0.829	0.755	0.852	0.740	0.771

**TABLE 5:** Comparison of fog detection performance on Daily Living dataset: The proposed HTSAN model achieves superior results (AUC = 0.88, F1 = 0.84) compared to Sigcha et al. (2022) and the top five models from Salomon and Gazit (2024).

Authors	AUC	F1	Accuracy	Precision	Recall
HTSAN (Proposed)	0.88	0.84	0.85	0.83	0.86
Sigcha et al. (2022)	0.65	0.71	0.66	0.62	0.83
1st Place (Salomon and Gazit, 2024)	0.850	0.777	0.860	0.733	0.826
2nd Place (Salomon and Gazit, 2024)	0.864	0.800	0.878	0.772	0.831
3rd Place (Salomon and Gazit, 2024)	0.843	0.768	0.855	0.726	0.815
4th Place (Salomon and Gazit, 2024)	0.838	0.761	0.852	0.724	0.803
5th Place (Salomon and Gazit, 2024)	0.829	0.755	0.852	0.740	0.771

**TABLE 6:** Comparison of fog detection performance on Hantao’s Multimodal dataset: The proposed HTSAN model achieves superior results (AUC = 0.96, F1 = 0.94) compared to Zhang L. et al. (2022), Bajpai et al. (2023) and Hou et al. (2023).

Authors	AUC	F1	Accuracy	Precision	Recall
HTSAN (Proposed)	0.96	0.94	0.98	0.93	0.95
Zhang et al. (2022b)	0.95	0.93	0.97	0.92	0.94
Bajpai et al. (2023)	0.86	0.73	0.86	0.73	0.73
Hou et al. (2023)	0.845	0.84	0.85	0.87	0.81

The HTSAN consistently outperformed other state-of-the-art models on all datasets. Thus, the presented model shows promising results for a variety of data types: real-world, multimodal, and task-induced. The contributions made by the use of CNN-based feature extraction, BiLSTM for temporal modeling, and the hybrid attention mechanism were quite high. Thus, this paper further shows HTSAN’s promise to improve freezing of gait detection and Parkinson’s disease monitoring.

### 4.7 Ablation studies

**FIGURE 13:**
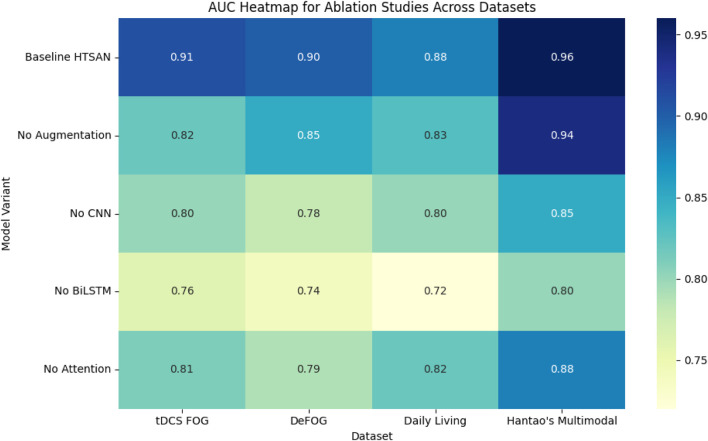
Impact of removing key components on performance metrics (accuracy and AUC) across multiple datasets, highlighting the importance of each component in overall model effectiveness.

To comprehensively evaluate the impact of key components in the proposed Freezing of Gait (FoG) detection framework, ablation experiments were conducted across four datasets: tDCS FOG, DeFOG, Daily Living, and Hantao’s Multimodal datasets (Figure 13). The focus was on Preprocessing (Augmentation), Spatial Feature Extraction, Temporal Sequence Modeling, and Attention Mechanism, aiming to determine the contribution of each component to the final performance, particularly the Area Under the Curve (AUC).

#### 4.7.1 Impact of preprocessing (augmentation)

Preprocessing, specifically Data Augmentation, was tested by disabling the augmentation step and using only the original datasets. Removing the augmentation from the training process led to a performance decline across all datasets. In the tDCS FOG Dataset, the AUC decreased from 0.91 to 0.82, highlighting how the absence of synthetic data limited the model’s ability to capture subtle gait variations effectively. Similarly, the DeFOG Dataset saw a reduction from 0.90 to 0.85, emphasizing the importance of diverse synthetic data for better generalization in noisy, real-world environments. The Daily Living Dataset also dropped from 0.88 to 0.83, showing how long-term datasets greatly benefit from improved diversity in data resulting from augmentation. The greatest drop was observed in the Hantao’s Multimodal Dataset, in which the AUC dropped from 0.96 to 0.94, signifying the critical role of augmented data when working with multimodal sensor inputs. The results obtained thus indicate that Data Augmentation is crucial for the improvement of robustness and performance, especially when the complexity or variability of real-world increases in datasets.

#### 4.7.2 Impact of spatial feature extraction

The importance of spatial feature extraction was tested by replacing the U-Net-inspired convolutional encoder with a standard CNN lacking skip connections and deeper feature extraction layers. For the tDCS FOG Dataset, it shows a trend of performance drops from 0.91 to 0.80, given that the standard CNN fails to carry fine-grained spatial features used for effective gait analysis. The effect would be more impactful in the case of the DeFOG Dataset wherein the AUC dropped from 0.90 to 0.78 for the need to have rich spatial features in noise environments.

A similar trend was also noticed in the Daily Living Dataset, where the AUC decreased from 0.88 to 0.80, indicating the need for retaining detailed spatial features for long-term gait recordings. In Hantao’s Multimodal Dataset, the AUC decreased from 0.96 to 0.85, which further shows the need for sophisticated spatial feature extraction when dealing with multimodal sensor data. The results confirm that the U-Net-based spatial extractor significantly improves detection accuracy by preserving fine details crucial for identifying subtle gait patterns.

#### 4.7.3 Impact of temporal sequence modeling

In order to evaluate the significance of temporal sequence modeling, the BiLSTM module was replaced with a standard 1D-CNN, which cannot learn temporal dependencies. The tDCS FOG Dataset was found to be decreased to 0.91 from a 0.76, while the DeFOG Dataset presented the most notable reduction, 0.90–0.74, since this latter dataset did not have a model of the time and had restricted the gait variations found in real worlds from being handled.

In the Daily Living Dataset, the AUC plunged from 0.88 to 0.72 while emphasizing the requirement of temporal models for longer observation periods. Also, the loss of the capacity to manage multimodal data made Hantao’s Multimodal Dataset have a decrease in AUC, from 0.96 to 0.80. These results confirm that BiLSTM layers are important for capturing both short-term and long-term dependencies, especially in datasets with extended gait recordings and real-world variability.

#### 4.7.4 Impact of attention mechanism

The effect of the attention mechanism was tested completely disabling it such that the model did not employ a focused strategy for attention. AUC in tDCS FOG Dataset changed from 0.91 to 0.81, while it indicated a little benefit coming from the inclusion of attention mechanisms even in a controlled dataset. A more prominent drop was shown by the DeFOG Dataset from 0.90 to 0.79, given that the presence of noisy gait patterns resulted in difficulty while dealing with noise without attention mechanisms.

For the Daily Living Dataset, where long-term sequential data is dominant, AUC dropped from 0.88 to 0.82, showing the importance of attention mechanisms in focusing on critical gait phases. The Hantao’s Multimodal Dataset showed the most significant drop, from 0.96 to 0.88, emphasizing the importance of attention when working with complex, multimodal sensor data. These results demonstrate that attention mechanisms significantly enhance performance, particularly in datasets with high variability, extended sequences, or multimodal complexity.

The ablation studies across all datasets reinforce the critical importance of each architectural component in the proposed FoG detection framework. Preprocessing (Data Augmentation) was essential for generalization, especially in complex datasets like DeFOG and Hantao’s Multimodal. The U-Net-based spatial extractor contributed significantly to improved accuracy by preserving detailed features crucial for FoG detection. Temporal sequence modeling, particularly using BiLSTM, proved critical in datasets with long-term gait recordings. Finally, the attention mechanism enhanced performance by focusing on essential gait phases and improving noise resilience. The experimental results demonstrate that the choice of dataset significantly affects model performance. Models trained on tDCS FOG performed best in controlled settings but required enhancements to generalize to real-world data. Attention and BiLSTM proved most effective for capturing both short-term and long-term gait patterns. Future work should focus on combining self-supervised learning with multimodal fusion to further improve generalization across datasets.

### 4.8 Comparative analysis

It states how the proposed FoG detection method is comprehensively compared with state-of-the-art models from recent literature, selected with relevance and based on performance to gait analysis using wearable sensor data (Table 7).

**TABLE 7:** Summary of Compared Methods for Freezing of Gait Detection. This table provides an overview of various methods used for FoG detection, highlighting their type, primary use case, key components, strengths, and limitations.

Method	Type	Primary use case	Key components	Strengths	Limitations
Mo and Chan (2023)	Deep Learning (CNN + LSTM)	Predicting three types of FOG events	CNN (Spatial Feature Extractor), LSTM (Temporal Modeling)	Effective at predicting multiple types of FOG events, good performance across various datasets	Requires extensive labeled data, computationally expensive
Sigcha et al. (2022)	Transformer based Deep Learning	FOG detection with accelerometers	Transformer Networks, Accelerometer Data	Excellent at handling noisy data and capturing long-range dependencies	Requires high computational power; complex training, Limited to waist-worn sensor data
Salomon et al. (2024) (1st–5th place results)	Machine Learning Contest	Automated FOG detection, time-of-day effects	Various ML approaches (e.g., ensemble models, CNNs, Transformers), wearable sensor data	Improved performance through competition, diverse algorithmic approaches	Variability in participant data, lack of external validation
1st Place (Salomon and Gazit, 2024)	Hybrid ML/DL	Best-performing model in contest	Combination of CNN, RNN, and handcrafted features	Highest accuracy in detecting FOG	High computational cost
2nd Place (Salomon and Gazit, 2024)	Deep Learning	Second-best model	Transformer-based architecture with feature extraction	Robust temporal modeling	Overfitting risks
3rd Place (Salomon and Gazit, 2024)	Ensemble Learning	Third-best model	Combination of decision trees, CNNs, and RNNs	Balanced precision-recall trade-off	Feature engineering required
4th Place (Salomon and Gazit, 2024)	Random Forest	Fourth-best model	Feature selection and RF classifier	Computationally efficient	Lower generalization ability
5th Place (Salomon and Gazit, 2024)	Statistical ML	Fifth-best model	Logistic regression with time-series analysis	Simple and interpretable	Limited complexity
Zhang et al. (2022b)	Multimodal Data Fusion	Detecting FOG in Parkinson’s Disease	EEG, IMU, and motion capture data integration	Multimodal analysis improves accuracy	Requires multiple sensor modalities
Bajpai et al. (2023)	Multimodal Data Fusion	Improved prediction of FOG	Fusion of audio, video, and sensor data	High robustness to noise, multimodal integration	Complex implementation, high data requirements
Hou et al. (2023)	Wireless Multi-Modal Sensors	Real-time FOG detection and alerting	Gel-free flexible sensors, edge computing, deep learning	Wearable, real-time, non-invasive detection	Edge AI limitations, battery constraints
Proposed Hybrid Temporal-Spatial Attention Network (HTSAN)	Deep Learning (CNN + BiLSTM + Attention)	FOG detection across multimodal datasets	CNN (Spatial Feature Extraction), BiLSTM (Temporal Modeling), Hybrid Attention Mechanism	Superior performance across multiple datasets, enhanced noise robustness, effective multimodal fusion	Computationally more expensive, requires multimodal data

These methods have been selected for their effectiveness in controlled and real-world FoG detection scenarios. Their diverse architectures and capabilities ensure a thorough comparison against the proposed model’s performance across different datasets and experimental conditions. Results have been analyzed from experimental work basing them on four datasets: tDCS FOG, DeFOG, Daily Living, and Hantao’s Multimodal. All these performances have been compared in terms of standard metrics such as AUC, accuracy, F1-score, precision, and recall. The results show variations for each dataset, relating to their complexity in the environment in which the data was collected, outlining both the robustness and limitations for each method. The critical analysis of the proposed work on different datasets is given in Table 4.

HTSAN, when applied across all four datasets, showed steady and significant improvements in performance relative to the other methods. This architecture, by incorporating CNN-based spatial feature extraction, BiLSTM sequence modeling, and a hybrid self-attention mechanism, made it easier to capture the sensor data both spatially and temporally. It enhanced the attention mechanism to further pay more focus to the significant gait phases of the models for better classification.

## 5 Conclusion

This paper introduces a hybrid deep learning approach designed to use wearable sensor data to identify FoG in individuals with Parkinson’s disease (PD). The integrated model achieves increased performance across several datasets by using CNNs for the extraction of spatial features, BiLSTM networks for temporal analysis, and an attention mechanism for better interpretability.

Evaluation on tDCS FOG, DeFOG, Daily Living, and Hantao’s Multimodal datasets proved the robustness and generalizability of the model, which showed significant improvements in accuracy, F1-score, and AUC compared to existing state-of-the-art methods. The study places a strong emphasis on advanced preprocessing techniques, including data augmentation, sensor fusion, and normalization, to address the challenges of noise, inter-subject variability, and imbalanced datasets. Ablation experiments further validated the critical contributions of individual model components, particularly the attention mechanism and temporal modeling, to enhance detection accuracy and robustness. The framework is optimized for real-time deployment and is thus well-suited for clinical and home-based applications, providing a scalable and reliable tool for FoG monitoring and management. Future research should focus on integrating self-supervised learning approaches and exploring multimodal data fusion to enhance the framework’s adaptability across varied environments and patient populations.

## Data Availability

The raw data supporting the conclusions of this article will be made available by the authors, without undue reservation.

## Footnotes

1 Photo Credit: https://neurologyassociatesva.com/warning-signs-of-parkinsons-disease-and-when-to-see-a-neurologist/
